# Self-reported breast and cervical cancer screening practices among women in Ghana: predictive factors and reproductive health policy implications from the WHO study on global AGEing and adult health

**DOI:** 10.1186/s12905-020-01022-5

**Published:** 2020-07-28

**Authors:** Martin Amogre Ayanore, Martin Adjuik, Asiwome Ameko, Nuworza Kugbey, Robert Asampong, Derrick Mensah, Robert Kaba Alhassan, Agani Afaya, Mark Aviisah, Emmanuel Manu, Francis Zotor

**Affiliations:** 1 School of Public Health, University of Health and Allied Sciences, University of Health and Allied Sciences, Ho, Ghana; 2 Institute of Health Research, University of Health and Allied Sciences, Ho, Ghana; 3 School of Nursing and Midwifery, University of Health and Allied Sciences, Ho, Ghana

**Keywords:** Breast and cervical screening practices, Adult women, Older women, Ghana

## Abstract

**Background:**

Breast and cervical cancers constitute the two leading causes of cancer deaths among women in Ghana. This study examined breast and cervical screening practices among adult and older women in Ghana.

**Methods:**

Data from a population-based cross-sectional study with a sample of 2749 women were analyzed from the study on global AGEing and adult health conducted in Ghana between 2007 and 2008. Binary and multivariable ordinal logistic regression analyses were performed to assess the association between socio-demographic factors, breast and cervical screening practices.

**Results:**

We found that 12.0 and 3.4% of adult women had ever had pelvic screening and mammography respectively. Also, 12.0% of adult women had either one of the screenings while only 1.8% had both screening practices. Age, ever schooled, ethnicity, income quantile, father’s education, mother’s employment and chronic disease status were associated with the uptake of both screening practices.

**Conclusion:**

Nationwide cancer awareness campaigns and education should target women to improve health seeking behaviours regarding cancer screening, diagnosis and treatment. Incorporating cancer screening as a benefit package under the National Health Insurance Scheme can reduce financial barriers for breast and cervical screening.

## Background

Breast and cervical cancers are major public health disease conditions globally [1–3]. An estimated 1.7 million breast cancer patients were recorded globally in 2012, accounting for about 25% of the world cancer burden among women [1]. Cervical cancer, another common form of cancer affecting women is linked to social inequality and poor standards of living, with mortality rates higher in developing countries [4].

In most developed countries, advances in medical imaging and technology has improved diagnosis and treatment for most cancers. This development led to a reduction in mortality levels and extended life expectancy among persons living with cancers [5, 6]. Pap test or HPV test, mammography, Magnetic Resonance Imaging (MRI), Clinical Breast Examination (CBE), Ultrasonography and Breast Self-Examination (BSE) have improved diagnosis and case detection for cervical and breast cancers [7, 8]. Cost of cancer treatments, poor health seeking habits and poor health infrastrature to provide diagnosis and treatment support are some reasons for high cancer death burdens in developing countries [9].

Cervical cancer mortality is two to three fold higher among women in developing countries, relative to women in developed countries [4]. A number of studies have shown that late presentation for screening and diagnosis, poor knowledge and awareness of the effects of breast and cervical cancer, socio-economic, cultural and other social factors are reasons why cancer mortality rates in developing countries are high [10, 11]. Studies have also shown that chronic conditions and its associations with breast and cervical cancers [12–14]. Breast and cervical screening practices are also found to be lower among persons with chronic illness [15].

In Ghana, breast and cervical cancers are increasingly investigated [16–18]. Breast cancer is reported to be on the increase and accounts for 15.4% of all cancer cases in the country [18, 19]. The overall breast cancer incidence in five regions was found to be 0.76%, with higher incidence among women aged 35 years and below [17]. Cervical cancer is also increasingly reported in Ghana, with about 3052 newly recorded cases and 1556 deaths in 2012 alone [20]. Despite the growing number of cancer cases in Ghana, cancer registries are absent, limiting the ability to contextualize the real scope of the magnitude of cancer burden in Ghana [17, 21]. Treatments for cancers in Ghana are also clinic-oriented, urbanized and often at a cost that cannot be paid by low income earners [19]. Other challenges impacting negatively on the burden of cancers in Ghana are the presentation of large clinical and histological advanced invasive cancers [20]. Survival rates among cancer patients in Ghana are reported to be low, largely due to adverse lifestyle determinants such as late presentation for diagnosis and care [19]. Evidence indicates that the burden of cancer in developing countries is likely to increase as most of these countries experience major demographic transitions over the next decades to come [21], indicating that current rates in Ghana will increase in the coming decades.

Population-based studies on prevalence, knowledge and practices on breast and cervical cancer are documented in Ghana [17, 22–24]. Other studies have found decreased quality of life among breast and cervical cancer patients in Ghana [25, 26], with all advocating for the need for timely screening, diagnosis and treatment. Despite the growing body of knowledge on breast and cervical cancer in Ghana, limited literature has examined and compared associations for breast and cervical cancer screening practices among adults (less than 50 years) and older women (50+ years). No study was identified when reviewing literature to have compared differences in screening practices between adult and older women in Ghana using national representative data. In addition, no study has examined effects of single or multiple chronic illnesses for breast and cervical cancers in Ghana. In addition, no study was found to have applied the WHO study on global AGEing and adult health (SAGE) Wave 1 data (2007–2008) in Ghana to compare breast and cervical screening practices and how chronic conditions impact on cancer screening practices. At the time of the study, although Wave 2 was conducted, the data were not publicly available on WHO website for use. To contribute to addressing the above knowledge gap and further understanding of factors associated with breast and cervical cancer in Ghana, this study examined the research question; what individual, household, and community level factors predict breast and cervical cancer screening practices among adult and older women in Ghana. The effects of single or multiple episodes of three chronic conditions; arthritis, angina and hypertension were included to assess their associations with breast and cervical screening practices. We applied the data from the WHO SAGE since we were interested in using a national representative sample. One study in South Africa was found to have applied the WHO SAGE data to explore correlates of breast and cervical screening practices [5]. No published study has applied the Ghana WHO SAGE Wave 1 data collected between 2007 and 2008 to examine correlates for breast and cervical cancer screening practices. Thus, the data remain relevant for informed decision making on individual, household, and community level factors that predict breast and cervical cancer screening practices among women of various age groups in Ghana. Specifically, this study finding adds to existing evidence of breast and cervical cancer screening practices along the reproductive life course. Policy lessons of what influences adult and older women’s screening practices would be useful in designing strategies that address each woman’s reproductive health care needs along the life continuum in Ghana.

## Methods

### Study design and data source

The study design was population-based cross-sectional. We analyzed data from the WHO study on global AGEing and adult health (SAGE) Wave 1 (2007–2008) in Ghana. WHO SAGE Wave 1 was conducted in six countries (China, Ghana, India, Mexico, Russian Federation and South Africa) led by WHO with national level collaboration. Wave 1 collected data among large samples of persons aged 50 years and older, and a smaller comparative group sample of 18–49 years. We extracted varaibles on disease risk factors, access to healthcare, health status and well-being among adult and older persons. For the purpose of this study, we adopted the proposed working definition of older persons from the minimum data set (MDS) Project that classified older persons to be aged 50 years and above [27].

### Sampling and population

The sampling method used for the Ghana SAGE Wave 1 was based on the design for the World Health Survey, 2003, in which the primary sampling units (PSUs) were stratified by region and location (urban/rural). Selection of the PSUs was based on proportional allocation by size. Each enumeration area (EA) was selected independently within each stratum [28]. In this study, we extracted only female data from the original dataset based on the aim of the study.

### Dependent variables

Two outcomes were assessed; breast and cervical screening practices. *To assess breast screening practices, the question, when was the last time you had a mammography if ever was applied. The variable was recoded as never had mammography = 0 (for those who had never had a mammography), and had a mammogram = 1 (for those who indicated the last time they had had a mammography).* The question, when was the last time you had pelvic examination, if ever? was recoded as *never had pelvic examination* = 0 (for those who had not previously had any pelvic examination), and *had a pelvic examination* = 1 (for those who indicated the last they had undergone pelvic examination). Also, an ordered variable (level of uptake) was derived from pelvic examination and mammography variables. This was recoded as *both pelvic examination and mammography* = 3 (High examination uptake), *either pelvic examination or mammography* = 2 (Low examination uptake), and *neither pelvic examination nor mammography* = 1 (No examination uptake).

### Independent variables

Age, marital status, religion, educational status, parent’s (father and mother) educational status, wealth status, employment status and parent’s (father and mother) employment status were the independent variables assessed. Age was regrouped as adults (< 50 years) =0 and older adults (50+ years) = 1, marital status regrouped as never married =1, currently married/cohabiting =2, widowed =3, separated/divorced = 4, while religion was regrouped as Christianity =1 and others (Islam, primal indigenous, Judaism, Buddhism) =0. Educational status was regrouped as formal education =1 and no formal education =0, employment status was regrouped as ever worked = 1, never worked = 0 and ethnicity regrouped as Akan = 1, Ewe =2, Ga-Adangbe =3, Gurma =4, and others (Grusi, Manda-Busanga, Mole-Dagbon, Guan) = 5. Co-variates to determine if respondents had arthritis, angina and hypertension were included to determine any potential associations with breast and cervical screening practices. Chronic disease is a barrier for breast and cervival cancer screening [13]. The type and number of chronic conditions of an individual also influence breast and cervical screening [12].

### Statistical analysis

Stata version 14.0 (StataCorp LLC) was used to analyse the data. Only variables of interest were extracted from the original SAGE wave 1 after access was provided by WHO to the principal investigator. First, descriptive analysis was conducted to assess the distribution of sociodemographic, Socio-Economic Status (SES) and breast (mammography) and cervical cancer (pelvic examination and pap smear) screening status among respondents. Binary logistic regression was used to assess associations between sociodemographic characteristics and breast and cervical cancer screening practices. Also, a multivariable ordinal logistic regression, specifically Proportional Odds Model (POM) was fitted to determine the factors associated with high levels of examinations uptake (Pelvic examination and Mammography (3) versus Pelvic examination or Mammography (2) and None (1) after the test for overall parallel assumption at 0.05 significance was upheld (*p* = .7105). Thus, the *p*-value (being higher than 0.05) indicates that the overall parallel assumption has not been violated. Statistical significance was based on *p* < 0.05. We excluded all missing values (MV) during the computation of association between the independent variables and the dependent variables.

## Results

*Socio-demographic characteristics of study respondents.*

Table 1 shows the socio-demographic characteristics of 2746 respondents who took part in the study. The mean age of study respondents was 61 years (SD: ±14), with 86.5% of respondents aged 50 years and above. A total of 54.8% respondents resided in rural areas. Regarding respondents marital status, 41.6% were widowed, 37.5% married/co-habiting, 17.5% were separated while 2.7% never married. About half of respondents (53.2%) had no formal education. Regarding income quintiles, about 18.7% were in 5th income category while 19.7% were in the 1st category. Close to half of respondents (45.2%) attended public health facility as their first point of seeking health care. Regarding screening practices for breast and cervical cancer, only 12.0% of women reported they had had a pelvic examination while 3.4% reported they had ever had a mammography (see Table 2). Only 1.8% of women had had both pelvic examination and mammography, 12.0% had had at least one while 86.2% had not undergone any of the examinations.

**Table 1:** Socio-demographic characteritics of study respondents

Characteristics		Frequency (%) ***N*** = 2746
Residence	Urban	1241 (45.2)
	Rural	1505 (54.8)
Age group (Mean (SD): 61(±14) years	< 50	368 (13.4)
	50 +	2376 (86.5)
	MV	2 (0.1)
Marital Status	Never	75 (2.7)
	Currently/cohabiting	1030 (37.5)
	Widowed	1142 (41.6)
	Separated/Divorced	481 (17.5)
	MV	18 (0.7)
Ever Schooled	No	1462 (53.2)
	Yes	945 (34.4)
	MV	339 (12.3)
Ethnicity	Akan	1326 (48.3)
	Ewe	174 (6.3)
	Ga Adangbe	261 (9.5)
	Gruma	124 (4.5)
	Others	488 (17.8)
	MV	373 (13.6)
Religion	Others	544 (19.8)
	Christian	1862 (67.8)
	MV	340 (12.4)
Income Quintile	1st	547 (19.9)
	2nd	565 (20.6)
	3rd	558 (20.3)
	4 ^th^	557 (20.3)
	5 ^th^	514 (18.7)
	MV	5 (0.2)
Mother ever employed	No	95 (3.5)
	Yes	2313 (84.2)
	MV	338 (12.3)
Mother’s education	No	2208 (80.4)
	Yes	173 (6.3)
	MV	365 (13.3)
Father ever employed	No	53 (1.9)
	Yes	2350 (85.6)
	MV	343 (12.5)
Father’s education	No	1881 (68.5)
	Yes	441 (16.1)
	MV	424 (15.4)
Health facility visited more often	Private facility	281 (10.2)
	Public facility	1240 (45.2)
	Charity facility	110 (4.0)
	pharmacy/Dispensary	323 (11.8)
	Other	145 (5.3)
	MV	647 (23.6)

MV-indicates missing values in the data. All missing data were excluded in the regression analysis

**Table 2:** Prevalence of pelvic and mammography examination among respondents

Characteristics		Frequency (%) ***N*** = 2746
Self-reported Pelvic Examination^a^ [*n* = 2390]	No	2103 (88.0)
	Yes	287 (12.0)
Self-reported Mammography^b^ [*n* = 2389]	No	2309 (96.6)
	Yes	80 (3.4)
Type of Examination performed^c^ [*n* = 2388]	None	2061 (86.2)
	Pelvic examination OR Mammography	287 (12.0)
	Pelvic examination AND Mammography	40 (1.8)

^a^Participants who had only pelvic examination

^b^Participants who had only mammography

^c^Participants who had either pelvic examination or mammogrpahy / participants who had both pelvic examination and mammography

### Factors associated with pelvic and mammography examination among respondents

The results of ordered logistic regression presented in Table 3 showed that older women aged 50 years and above were 44% less likely to perform both pelvic and mammography examinations, compared to women aged below 50 years (aOR = 0.66, 95% CI 0.44–0.89). Women belonging to the highest income quintile (5th) were 68% more likely to perform both pelvic examination and mammography examinations (aOR = 1.68, 95% CI 1.04–2.71). Respondent’s mother’s educational status was associated with women performing both pelvic and mammography examinations (aOR = 0.33, 95% CI 0.58–1.48). Respondent’s educational status and their father’s educational attainments were also statistically associated with performing both pelvic examination and mammography examinations respectively (aOR = 1.44, 95% CI 1.05–1.98) and (aOR = 1.70, 95% CI 1.20–2.40).

**Table 3:** Associations between odds of pelvic and mammography examination uptake and socio-demographic characteristics of respondents

Characteristics		Binary logistic regression		Ordered logistic regression
		Pelvic Examination	Mammography	Pelvic Examination AND Mammography
		COR (95% C.I)	COR (95% C.I)	AOR (95% C.I)
Residence	Urban (ref)			
	Rural	0.61 (0.48–0.78) ***	0.65 (0.42–1.02)	1.10 (0.81–1.50)
Age group	< 50 years (ref)			
	50 + years	0.49 (0.36–0.65) ***	0.47 (0.28–0.78) **	0.66 (0.45–0.98) *
Marital Status	Never (ref)			
	Currently cohabiting	0.73 (0.38–1.40)	0.46 (0.17–1.23)	0.70 (0.35–1.41)
	Widowed	0.45 (0.23–0.87) *	0.27 (0.10–0.72) *	0.59 (0.28–1.23)
	vSeparated/Divorced	0.72 (0.36–1.41)	0.59 (0.22–1.63)	0.87 (0.42–1.82)
Ever Schooled	No (ref)			
	Yes	2.86 (2.22–3.69) ***	2.67 (1.68–4.23) ***	1.44 (1.05–1.98) *
Ethnicity	Akan (ref)			
	vEwe	1.46 (0.96–2.23)	1.01 (0.42–2.40)	1.48 (0.93–2.34)
	Ga Adangbe	1.12 (0.77–1.65)	1.13 (0.56–2.28)	1.15 (0.76–1.73)
	Gruma	1.46 (0.89–2.37)	1.44 (0.60–3.44)	1.10 (0.62–1.94)
	Others	0.37 (0.24–0.57) ***	0.73 (0.38–1.38)	0.53 (0.32–0.88) *
Religion	Others (ref)			
	Christian	2.26 (1.57–3.27) ***	2.68 (1.28–5.60) **	1.02 (0.65–1.60)
Income Quintile	1 (ref)			
	2	0.93 (0.58–1.48)	1.56 (0.60–4.06)	0.86 (0.52–1.42)
	3	1.28 (0.83–1.98)	1.59 (0.61–4.13)	1.09 (0.68–1.75)
	4	1.85 (1.23–2.79) **	3.61 (1.55–8.43) **	1.43 (0.90–2.27)
	5	2.69 (1.81–4.02) ***	4.22 (1.81–9.83) **	1.68 (1.04–2.71) *
Mother ever employed	No (ref)			
	Yes	0.26 (0.16–0.40) ***	0.41 (0.18–0.91) *	0.33 (0.18–0.60) ***
Mother’s education	No (ref)			
	Yes	2.16 (1.47–3.19) ***	2.59 (1.40–4.79) **	0.93 (0.58–1.48)
Father ever employed	No (ref)			
	Yes	0.31 (0.17–0.58) ***	0.84 (0.20–3.50)	0.51 (0.22–1.15)
Father’s education	No (ref)			
	Yes	3.05 (2.32–4.01) ***	2.84 (1.78–4.55) ***	1.70 (1.20–2.40) **
Health facility visited more often	Private facility (ref)			
	Public facility	0.67 (0.47–0.95) *	1.45 (0.68–3.09)	0.94 (0.64–1.37)
	Charity facility	0.90 (0.50–1.63)	1.63 (0.52–5.08)	1.31 (0.70–2.44)
	Pharmacy	0.31 (0.18–0.53) ***	0.87 (0.32–2.35)	0.60 (0.35–1.05)
	Other	0.54 (0.29–0.99) *	1.48 (0.50–4.36)	0.94 (0.50–1.76)
Self-reported arthritis	No (ref)			
	Yes	0.57 (0.38–0.87) **	1.03 (0.55–1.93)	0.76 (0.50–1.14)
Self-reported angina	No (ref)			
	Yes	0.78 (0.39–1.57)	1.31 (0.47–3.67)	0.79 (0.36–1.72)
Self-reported hypertension	No (ref)			
	Yes	1.64 (1.21–2.21) **	2.05 (1.24–3.39) **	1.13 (0.79–1.62)

Ref: Reference category *p < 0.05, **p < 0.01, ***p < 0.001

## Discussion

### Overview of socio-demographic findings

This study explored breast and cervical cancer screening practices and their associated factors among Ghanaian women using national representative data from the WHO SAGE Wave 1 conducted in 2008. Our findings show close to half of respondents (41.6%) were widowed. In comparing this study to similar SAGE Wave 1 studies conducted in China, Mexico, India, South Africa and Russia within the same period (2007–2010), more adult and older women in Ghana were widowed, relative to other SAGE country findings examined [29]. This study also found more women to belong to the poor and low socio-economic groups, as a high number of respondents were within the 1st to 3rd income quintiles, indicating a high number of respondents belonged to the lower socio-demographic index (SDI). Studies corroborate income quintiles [30, 31] and socio-economic status [32, 33] as predictors of breast and cervical cancer screening practices. Poor wealth status is also evident as a risk factor that impacts on poor equity outcomes on breast and cervical cancer screening practices among women [34]. Studies have examined that health insurance plays a mediating role in removing financial barriers to breast and cervical screening practices in some settings [35].

In comparing our study findings on income quintile to another WHO SAGE country study in South Africa, we found similar results regarding the respondents in the 1st to 3rd income groups, relative to respondents in other income groups [5]. The findings on income groups are important to explain for any current screening practices regarding breast and cervical cancer at the population level. Breast and cervical screening practices in China, Mexico, Russia and South Africa correlates with income levels [35]. In addition, type of health facility, the time of presentation for cancer diagnosis and treatment are linked to wealth status in many primary level studies in sub-Saharan Africa [36].

### Self-reported breast and cervical screening practices

More women in this study were found to have had breast examination compared to women who had ever screened for cervical cancer. Our analysis found 12.0% of women to have ever undergone pelvic screening while only 3.4% of women had ever received a pelvic examination and pap smear. A previous study in Ghana found 2% of women to have ever had a mammography in the past in two cities [37]. This is lower compared to these study findings. The differences could potentially be mediated by varied socio-demographic variables across the two sample populations. Other studies [5, 38, 39] corroborate the lower rate of breast and cervical cancer screening practices found in our study, and the potential role of social factors accounting for this trend.

In Ghana, despite growing concerns of cancer mortality rates and the commitment by successive Ghanaian governments to improve awareness of breast and cervical cancer screening practices [40], low awareness on the etiology of cancers in Ghana among the public could still explain for low screening practice rates as found in this study. Other studies that have reported low screening rates for either breast or cervical screening practices in Ghana cite poor knowledge of prevention and treatment of cancers and poor behavioral attitudes [39, 41]. The lack of organized national screening centres also accounts for this low rate of screening practices in Ghana [42]. An estimated 2.7% of women screen for cervical cancer regularly in Ghana [24]. This evidence could however be underestimated in Ghana due to the lack of a national cancer registry.

At the policy level in Ghana, there has been concerted effort by the Ministry of Health to improve population awareness and behavioral practices with regards to all **forms** of cancers affecting the population [43]. To improve two strategic objectives outlined by the Ghana Ministry of Health [40] for cancer control; increase awareness and uptake of screening programs and deploy organized and other opportunistic screening strategies in Ghana, there is a need for health sector policies to invest in building robust and reliable data system that can provide vital statistics on the burden of cervical and breast cancer needs in Ghana. Alongside this, long term financing for mass screening for women that would take away any financial challenges by means of Ghana’s National Health Insurance Scheme (NHIS) could improve behavioural practices and late presentations for screening for most cancers in Ghana.

Comparing the rates for both breast and cervical screening practices in this study to other African countries, one study among women in Gabon, Central Africa found higher cervical cancer screening practices among respondents after recommendations from a doctor to undergo screening [44]. Differences in screening rates between this study and the Gaboniase study could be due to the recommedation for patients to seek screening as a follow-up for clinical diganosis for cancer, indicating that public health policies by medical professionals for women to screen for breast and cervical practices could improve population level uptake and behavioural practices on cancer screening. Mammography examination was also reported to be low in other SAGE country findings [45], similar to what is found in Ghana in this study. However, a relatively high rate (15.5%) of respondents in South Africa reported they had ever had mammography examination [5]. Country differences regarding socio-economic status, access to health infrastructure, and population level seeking behaviours could explain for the differences in high rates in the South African population, relative to Ghana and other countries in Africa.

### Correlates of breast and cervical cancer screening practices among Ghanaian women

We found older women (50+ years) to be **less likely to have received both** breast and cervical cancer examinations, compared to adult women less than 50 **years**. Similar evidence has been corroborated in the World Health Survey that found that women within the younger age group were 8 times more likely to perform cervical cancer test (Pelvic exam/pap smear) than women in the older age group [45]. This age difference in both breast and cervical cancer screening practices may reflect inequalities in health services as most of these preventive healthcare services are targeted at younger adult women, with the potential to miss women aged 50+ years. Thus, younger adult women (< 50 years) may benefit from such these preventive healthcare practices than older females. One other possible explanation for younger women’s increased likelihood to receive breast and cervical screening is due to higher education and awareness of the potential risk of cancers, compared to older women (> 50 years). This corroborates a study conducted in Egypt that found that younger women were at higher risk of breast cancer than older women [46].

Also, the odds of receiving either breast or cervical exams is higher among women who had ever schooled than those who never schooled as corroborated by this study. Schooling has the potential to increase life earnings and individual health seeking behaviours in the life course [33]. Being educated in school could influence women’s understanding of the burden of cancer-related diseases and its prevention methods. This may be due to the fact that most of health promotion programmes use schools as avenues and persons who have ever been to school may appreciate the burden of both breast and cervical cancer than persons with no formal education. However, a cross-sectional study conducted in Iran showed that educated women were less likely to receive breast cancer examination [47]. This difference could be as a result of study location and culture.

Socio-economic status (SES) was a strong determinant for young adult and older women to report ever having been screened for breast and cervical cancer. The odds of screening increases with increasing wealth to the largest wealth group (5th income quintile), **compared** to poorer income group (1st income quintile). Studies on breast and cervical screening corroborates what was found in this study regarding socio-economic status effects on breast and cervical cancer screening practices [5, 45]. A review on early methods of breast cancer detection revealed mammography in low middle income countries is demanding in human and financial resources [48]. Also, screening for breast cancer involves complex infrastructure that may not be feasible in many low- and middle-income countries [49], an evidence that could also explain why this study found low rates for screening for breast and cervical cancer in Ghana.

We also found ethnicity, mother’s employment status, and father’s educational status to be associated with breast and cervical cancer screening practices in this study, further corroborating studies that have found similar results [33, 48, 50]. In the present study, ethnic minorities (grouped as “Others”) were less likely to have high uptake of pelvic examination and mammography. Our finding is indicative of the possible existence of cultural beliefs which ultimately influence health seeking behaviour. Father’s education had a positive association with uptake. This may be due to the fact that educated fathers may influence their daughters or partners to go for screening since they may be aware of the benefits thereof. Mothers who were ever employed are more likley not to have ever had pelvic and breast screening. The reason is that employed mothers may be too busy and may not have ample time to go for screening. In assessing for chronic conditions and their effects on breast and cervical screening in this study, our findings showed that a significant association exists between chronic condition (hypertensive women and angina conditions) and breast and cervical screening practices. This finding corroborates evidence established in literature on chronic illness and its lowered effects on cancer screening interventions [12–15].

This study has some limitations. Missing values that could not be explained were excluded in our analysis. Interpretations of rates should therefore take recognition of these shortfalls in the data. The use of secondary data also limits our ability to draw strong causal links regarding our findings. Future studies that draw on much broader national level scope and based on well functioning cancer registry database will be helpful in addressing data reliability issues on this topic in Ghana.

### Policy relevance and health systems readiness in the context of study findings

Self-reported breast screening and Pap smears for cervical cancer screening are a good marker of the health system’s response to women’s health in the country [51]. The low rate of screening practices among women in this study raises concerns about the readiness of the present health system to address this significant backdrop. Although a national programme for the control of non-communicable disease exists, much has not been done with regard to breast and cervical cancer. Among the main policy and programmatic challenges of the cancer control plan are low political interest, inadequate funding, inept programme management, low community awareness, and absence of organized screening programmes [52]. A renewed political commitment is therefore essential to combat these two cancers that are the leading causes of mortality from cancer among women in Ghana.

The cost of screening also renders some women disadvantaged, especially older women who may not be working [4, 29]. Consistent governmental funding of a national cancer screening programme would be a sure step towards early detection and treatment of breast and cervical cancer. This would help subsidize the cost of screening for women and remove the barrier of low income associated with the uptake of screening services. Inclusion of screening services under the National Health Insurance Scheme as part of efforts to improve current rates of screening practices could also counterbalance the financial barriers to the uptake of screening services. In addition, funding is needed to scale up preventive education and awareness creation above the status quo, and set up more centres where screening can be accessed.

As stated in Ghana’s policy for the prevention and control of non-communicable diseases, breast and cervical cancer screening were supposed to be integrated into reproductive health services [40]. It is however not so in most health facilities across the country due to some of the programmatic challenges mentioned earlier [52]. A formal integration of screening services into the already existing health system is recommended. This can help improve current rates of screening practices and reduce the lifetime risk of these cancers.

## Conclusion

In this study, the uptake of breast and cervical cancer examination among adult and older Ghanaian women was influenced by age, educational status, ethnicity, and level of income. In line with ensuring high breast and cervical cancer screening practices and healthy living among women in Ghana, there is an urgent need for nationwide cancer awareness campaigns to improve knowledge. Awareness campaigns would be very instrumental in closing acceptability of cancer screening and practices gaps. Given that susceptibility to cancer increases with age, and vulnerable groups are likely to suffer the effects of poor cancer screening, diagnosis and treatment, nationwide cancer awareness campaigns and education interventions should target both the aged (50+ years) and younger adult women to improve on health seeking behaviours regarding cancer screening, diagnosis and treatment. Investments in vital data systems and cost-effective strategies to ensure that cost is not a barrier to screening and care are long overdue.

## Data Availability

All data applied in this study is available upon request online from the WHO website.

## Abbreviations

MRI: Magnetic Resonance Imaging
CBE: Clinical Breast Examination
BSE: Breast Self-Examination
MDS: Minimuim Data Set
PSU: Primary Sampling Units
MV: Missing Values
SAGE: study on global AGEing and Adult Health
SD: Standard Deviation
SDI: Socio-demographic Index
MoH: Ministry of Health
NHIS: National Health Insurance Scheme
SES: Socio-economic Status
WHO: World Health Organization
